# Kinetics of cytochrome P450 enzymes for metabolism of sodium tanshinone IIA sulfonate in vitro

**DOI:** 10.1186/s13020-016-0083-z

**Published:** 2016-03-22

**Authors:** Dong-sheng Ouyang, Wei-hua Huang, Dan Chen, Wei Zhang, Zhi-rong Tan, Jing-bo Peng, Yi-cheng Wang, Ying Guo, Dong-li Hu, Jian Xiao, Yao Chen

**Affiliations:** Department of Clinical Pharmacology, Xiangya Hospital, Central South University, 110 Xiangya Road, Changsha, 410078 Hunan China; Institute of Clinical Pharmacology, Central South University, 110 Xiangya Road, Changsha, 410078 Hunan China; Department of Pharmacy, Xiangya Hospital, Central South University, 87 Xiangya Road, Changsha, 410008 Hunan China

## Abstract

**Background:**

Sodium tanshinone IIA sulfonate (STS) is a water-soluble derivative of tanshinone IIA for treating cardiovascular disorders. The roles of cytochrome P450 enzymes (CYPs) in the metabolism of STS have remained unclear. This study aims to screen the main CYPs for metabolism of STS and study their interactions in vitro.

**Methods:**

Seven major CYPs were screened for metabolism of STS by human liver microsomes (HLMs) or recombinant CYP isoforms. Phenacetin (CYP1A2), coumarin (CYP2A6), tolbutamide (CYP2C9), metoprolol (CYP2D6), chlorzoxazone (CYP2E1), S-mephenytoin (CYP2C19), and midazolam (CYP3A4) were used as probe substrates to determine the potential of STS in affecting CYP-mediated phase I metabolism in humans. Enzyme kinetic studies were performed to investigate the modes of inhibition of the enzyme–substrate interactions by GraphPad Prism Enzyme Kinetic 5 Demo software.

**Results:**

Sodium tanshinone IIA sulfonate inhibited the activity of CYP3A4 in a dose–dependent manner by the HLMs and CYP3A4 isoform. The *K*_*m*_ and *V*_*max*_ values of STS were 54.8 ± 14.6 µM and 0.9 ± 0.1 nmol/mg protein/min, respectively, for the HLMs and 7.5 ± 1.4 µM and 6.8 ± 0.3 nmol/nmol P450/min, respectively, for CYP3A4. CYP1A2, CYP2A6, CYP2C9, CYP2D6, CYP2E1, and CYP2C19 showed minimal or no effects on the metabolism of STS.

**Conclusion:**

This in vitro study showed that STS mainly inhibited the activities of CYP3A4.

**Electronic supplementary material:**

The online version of this article (doi:10.1186/s13020-016-0083-z) contains supplementary material, which is available to authorized users.

## Background

**Fig. 1:**
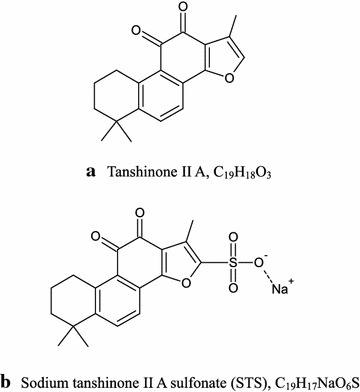
Chemical structures of tanshinone IIA (**a**) and STS (**b**)

Tanshinone IIA (Fig. 1a) is one of the main extracts from *Salvia miltiorrhiza* (*Danshen*) for treating cardiovascular disorders [1]. Tanshinone IIA is the most effective fat-soluble monomer extracted from *Danshen*, with anti-inflammatory [2], antitumor [3], antioxidative [4, 5], and antiplatelet aggregation activities [6, 7]. However, as clinical use of tanshinone IIA is limited by its poor water solubility, sodium tanshinone IIA sulfonate (STS) (Fig. 1b) has been developed through sulfonation with greater water-soluble characteristics and efficacies than tanshinone IIA [8], and has been used in injection with typical doses of 40–80 mg/d for cardioprotective [9, 10], anti-cardiomyocyte hypertrophy [11], and antiviral effects [12–14]. To date, only several studies reported the pharmacokinetics parameters of STS in rats after injection. The metabolism rate of STS in rats was fast, with t_1/2_ of <0.9 h, and at 4 h after the injection, the plasma concentration of STS in rats did not exceed 20 ng/mL [15, 16].

CYPs comprise the most important phase I drug-metabolizing enzyme system, and are responsible for the metabolism of a variety of xenobiotics [17]. Yang et al. [18] reported that tanshinone (containing 20 % tanshinone IIA) could significantly increase (2–9.5-fold) the activities of CYP1A1, CYP1A2, and CYP2B1, while inhibiting (1.9-fold) the activity of CYP2E1 in rats. Ueng et al. [19] reported that tanshinone IIA could decrease 7-ethoxyresorufin O-deethylation (EROD) and 7-methoxyresorufin O-demethylation (MROD) activities in human liver microsomes (HLMs), and indicated that tanshinone IIA possessed the highest selectivity for inhibition of CYP1A2.The same authors further demonstrated that tanshinone IIA induced hepatic Cyp1A2 by increasing the expression levels of Cyp1A2 mRNA and protein in mice [20]. Liu et al. [21] reported that CYP2A6 was the specific isozyme responsible for hydroxyl metabolism of tanshinone IIA in HLMs. Although many experiments related to tanshinone IIA have been performed, the question of which CYP is responsible for the metabolism of STS has remained unclear.

The difference in molecular structure between STS and tanshinone IIA is only a sulfonic acid group at the C-16 position [22]. Although STS has a similar molecular structure to tanshinone IIA, the metabolism of STS and interactions with CYPs might differ from those of tanshinone IIA [16]. In our previous study, STS significantly increased the activity of CYP1A2 by 41.1 % in healthy volunteers [23], and the possible mechanism might be the induction of CYP1A2 expression by tanshinone IIA transformed from STS in vivo. Which CYP participated in the transformation of STS, and related information is still critical.

As CYP1A2 (13 %), CYP2A6 (4 %), CYP2D6 (2 %), CYP2E1 (7 %), CYP3A4 (30 %), CYP2C19 (8 %) and CYP2C9 (20 %) comprise 70–80 % of the hepatic CYPs in humans and are responsible for more than 95 % of clinical drug metabolism [17]. This study aims to screen the main CYPs for metabolism of STS and study their interactions in vitro.

## Methods

### Enzymes and chemicals

Recombinant human CYP enzymes (bactosomes), pooled human liver microsomes (HLMs) from ten individual donors (bactosomes), and β-nicotinamide adenine dinucleotide 2′-phosphate reduced tetrasodium salt hydrate (NADPH) were purchased from Cypex Ltd. (UK) and stored at −80 °C. STS (C_19_H_17_NaO_6_S; MW: 396.39; assay: ≥98 %) was obtained from Sigma-Aldrich (China). Furafylline (FUR), trans-2-phenylcyclopropylamine hydrochloride (TRA), ketoconazole (KET), sulfaphenazole (SUL), quinidine (QUI), chlormethiazole hydrochloride (CHL), and ticlopidine hydrochloride (TIC) were purchased from the National Institutes for Food and Drug Control (China). Phenacetin, coumarin, midazolam, tolbutamide, S-mephenytoin, metoprolol, chlorzoxazone, and standards for their metabolites, including acetaminophen, 7-hydroxyl coumarin, 1-hydroxyl midazolam, 4-hydroxyl tolbutamide, 4-hydroxyl mephenytoin, α-hydroxyl metoprolol, 6-hydroxyl chlorzoxazone, and irbesartan (internal standard) were purchased from Sigma-Aldrich (China). All other chemicals and solvents were of high-performance liquid chromatography (HPLC) grade.

### Apparatus and operation conditions

The concentrations of the CYP substrates and their metabolites were quantified using a Waters 2695 Separation Module HPLC System (Waters Corp., USA) coupled to a Quattro Micro™ API Triple Quadrupole Tandem Mass Spectrometer (Waters Corp., USA) with an electrospray ionization source. The samples were separated on a HYPURITY C_18_ column (150 × 2.1 mm; internal diameter: 5 µm; Thermo, USA) with a C_18_ security guard column (4.0 × 3.0 mm; internal diameter: 5 µm). The mobile phase consisted of 20 mM ammonium formate and acetonitrile at a ratio of 60:40. Aliquots of 20 µL were injected at a mobile phase flow rate of 0.3 mL/min. Multiple reaction monitoring was performed in positive or negative modes according to different compounds. The transitions were listed in Table 1. The mass spectra of the metabolites formed in the incubations were identical to those of the corresponding authentic standards, including 7-hydrxoyl coumarin, 1- hydroxyl midazolam, 4- hydroxyl tolbutamide, 4- hydroxyl mephenytoin, α- hydroxyl metoprolol, 6- hydroxyl chlorzoxazone [24].

**Table 1:** Transitions and collision energies used in LC-MS/MS for the detection of the probe substrates, metabolites and the internal standard

Compound name	Ion mode	Precursor ion (m/z)	Product ion (m/z)	Collision energy (eV)
Sodium tanshinone IIA sulfonate	Negative	373	358	20
Acetaminophen	Positive	152	110	15
7-hydroxyl coumarin	Positive	162.9	107.0	20
1-hydroxyl midazolam	Positive	342	324	20
4-hydroxyl tolbutamide	Positive	287.0	171.0	15
4-hydroxyl mephenytoin	Positive	235.0	150.0	10
α-hydroxyl metoprolol	Positive	284.3	116.0	20
6-hydroxyl chlorzoxazone	Positive	195	138	20
Irbesartan (IS)	Positive	429.0	206.9	22

### Incubation conditions

The inhibitory effects of STS on the activities of the CYP isoforms were examined by HLMs (and the expressed CYPs, when required). The CYP isoform-specific probe reactions used were phenacetin O-deethylation (for CYP1A2), coumarin 7-hydroxylation (for CYP2A6), tolbutamide 4-hydroxylation (for CYP2C9), metoprolol α-hydroxylation (for CYP2D6), chlorzoxazone 6-hydroxylation (for CYP2E1), S-mephenytoin 4-hydroxylation (for CYP2C19), and midazolam 1-hydroxylation (for CYP3A4). The incubation mixtures consisted of substrate probe, HLMs (0.5 mg/mL) or CYP isoforms (10 pmol), and 0.1 M sodium phosphate buffer (pH 7.4) in a total volume of 0.2 mL that was pre-warmed for 5 min at 37 °C without (control) and with multiple concentrations of STS. Each reaction was initiated by addition of 1 mg/mL NADPH. The final incubations were performed in a shaking water bath at 37 °C for 30 min. The incubations were performed in triple, and the incubation conditions specific to each CYP isoform were within the linear range for the velocity of the reaction (incubation time as well as substrate and protein concentrations). STS, probe substrates, and inhibitors were dissolved in methanol, and the final solvent concentration in all incubations (including controls) was 1 %. The reactions were stopped by adding 0.2 mL of ice-cold acetonitrile containing irbesartan (114.9 ng/mL) as the internal standard. The samples were vortexed for 5 min. After centrifugation (5415D centrifuge, Eppendorf, Germany) (12,000×*g* for 10 min), the supernatants were transferred and aliquots of 20 µL were injected into the HPLC–MS/MS system for analysis.

### Kinetic analysis of STS

Kinetic analyses were performed for STS, and the data generated were used as a guide for selecting the appropriate concentrations of STS in the subsequent inhibition experiments. Thus, the kinetic parameters for the metabolism of STS were determined by incubating increasing concentrations of STS (1–100 µM) (without inhibitor) at 37 °C with HLMs (0.5 mg/mL) or CYP isoforms (10 pmol), and 0.1 M sodium phosphate buffer (pH 7.4) in a total volume of 0.2 mL. The incubation conditions consisted of substrate probe, HLMs (0.5 mg/mL) or CYP isoforms (10 pmol), and 0.1 M sodium phosphate buffer (pH 7.4) in a total volume of 0.2 mL. The equation for STS reaction velocity (*V*) by the HLMs or CYP isoforms was expressed as *V* = *(C*_*0*_−*C*_*t*_*)/*T*/C*_*p*_, where *C*_*0*_ and *C*_*t*_ represent the initial and final concentrations of STS in the incubation solution, respectively, T is the incubation time (min), and *C*_*p*_ is the protein concentration (mg/mL or nmol). All values were expressed as the mean ± standard deviation (SD). The mean intrinsic clearance rate (*CL*_*int*_) for the in vitro incubation was estimated by *V*_*max*_*/K*_*m*_.

### Specific CYP isoforms screened for metabolism of STS

We determined the inhibitory effects of specific inhibitors on the metabolism of STS by HLMs to screen for the specific CYP isoform responsible for STS metabolism. Inhibitors including FUR (CYP1A2 inhibitor; 1 µM), TRA (CYP2A6 inhibitor; 1 µM), SUL (CYP2C9 inhibitor; 1 µM), QUI (CYP2D6 inhibitor; 1 µM), CHL (CYP2E1 inhibitor; 5 µM), TIC (CYP2C19 inhibitor; 1 µM), and KET (CYP3A4 inhibitor; 1 µM) were separately incubated with STS (10 µM), HLMs, and NADPH under the same incubation conditions as mentioned above. The concentrations of the inhibitors used were approximately the same as their respective *IC*_*50*_ values from a previous report [25]. The inhibitory effects of the specific inhibitors on the metabolic clearance rate (MCR) of STS were evaluated separately to screen for the CYP isoforms responsible for STS metabolism. The relative activity of each CYP isoform was calculated by dividing the peak area of STS incubated with the inhibitor by the peak area of STS in the negative control.

### Inhibition studies for *IC*_*50*_ determination

A pilot inhibitory analysis of each CYP isoform was performed to determine the potency of inhibition and to select CYP isoforms for further detailed study of their inhibitory activities. Various concentrations of STS (1–100 µM) and a single CYP isoform-specific substrate concentration (the *K*_*m*_ value) were used to determine the inhibitory effects of STS on specific CYP isoforms. The substrates comprising phenacetin, coumarin, tolbutamide, metoprolol, chlorzoxazone, S-mephenytoin, and midazolam were employed at concentrations of 10, 5, 100, 7.5, 40, 100, and 5 µM, respectively [25]. All incubation conditions were the same as those described above. The inhibitory effects on the CYP isoforms were investigated individually by incubating HLMs in the absence or presence of STS. An incubation solution containing only the solvent used to dissolve STS was regarded as the negative control, while solutions containing only the specific inhibitors were regarded as positive controls. The *IC*_*50*_ values for STS were determined and compared with those of the specific inhibitors mentioned above (Table 2).

**Table 2:** *IC*_*50*_ and *K*_*i*_ values of STS against human CYP isoforms compared with that of specific inhibitors reported in literature

CYP	Activity	*IC*_*50*_ (µM)		*K*_*i*_ (µM)	
		STS	Specific inhibitor/reported values^a^	STS	Specific inhibitor/reported values^a^
CYP1A2	Phenacetin O-deethylation	>100	FUR/1.4 [23]	–	FUR/3 [21]
CYP2A6	Coumarin 7-hydroxylation	>100	TRA/0.42 ± 0.07 [24]	–	TRA/0.17 [24]
CYP2C9	Tolbutamide 4-hydroxylation	>100	SUL/0.3–1.5 [23, 25]	–	SUL/0.3 [21]
CYP2D6	Metoprolol α-hydroxylation	>100	QUI/0.02–0.68 [23, 25]	–	SUL/0.027–0.4 [21, 26, 27]
CYP2E1	Chlorzoxazone 6-hydroxylation	>100	DIE/21.30 [23]	–	CHL/12 [28]
CYP2C19	S-Mephenytoin 4-hydroxylation	>100	TCL/0.52–1.6 [25]	–	TCL/1.2 ± 0.5 [21]
CYP3A4	Midazolam 1-hydroxylation	6.377 (5.536, 7.347)^b^	KET/0.08–0.24 [21]	3.183 (0.184, 6.95)^b^	KET/0.015 [21]

“–” Represents the data that is not determined

*STS* sodium tanshinone II A sulfonate; *FUR* furafylline; *TRA* trans-2-phenylcyclopropylamine hydrochloride; *SUL* sulfaphenazole; *QUI* quinidine; *CHL* chlormethiazole hydrochloride; *TIC* ticlopidine hydrochloride; *KET* ketoconazole; *DIE* diethyldithiocarbamate

^a^ *IC*_*50*_ and *K*_*i*_ values of specific inhibitors were referred to the reported literatures

^b^ Represents 95 % confidence interval

### Determination of *K*_*i*_

In pilot experiments (*IC*_*50*_ determination), STS inhibited CYP3A4, while its effects on the remaining CYPs (CYP1A2, CYP2A6, CYP2C9, CYP2D6, CYP2E1, and CYP2C19) were minimal. Therefore, Dixon plots for the inhibition of CYP3A4 were determined by incubating the substrate probe at multiple concentrations with or without the test inhibitor at multiple concentrations with the HLMs and cofactors. The inhibition data obtained from the pilot experiments were used as a guide to generate appropriate probe substrate and test inhibitor concentrations for determination of the *K*_*i*_ values for each CYP isoform. The isoform-specific probe substrate concentrations used were 5–50 µM midazolam for CYP3A4. The STS concentrations used were 0–100 µM.

### Calculation of enzyme kinetics and statistical analysis

The MCR of the incubation solution without any specific inhibitor for STS was considered to be 100 % to determine the major enzymes responsible for STS metabolism in HLMs. The effects of the specific inhibitors on the MCR of STS were evaluated by one-way analysis of variance (ANOVA) (SPSS Inc., USA), LSD test was used for the homogeneity of variance, otherwise Tamhane’s test. Values of *P* < 0.05 denoted significance in all cases. The apparent kinetic parameters of STS (*K*_*m*_ and *V*_*max*_) were determined by fitting the Michaelis–Menten equation using GraphPad Prism Enzyme Kinetic 5 Demo software (GraphPad Co. Ltd., USA). The equation was expressed as *V* = *V*_*max*_*[S]/(K*_*m*_ + *[S])*,where *K*_*m*_ is the substrate concentration at which the reaction velocity is 50 % of *V*_*max*_. The activity of each CYP isoform was calculated by the MCR of its corresponding probe substrate to determine the inhibition of the CYP isoforms. The MCR of the probe substrate was considered to be 100 % when no specific inhibitor and STS were added to the incubation assay. The *IC*_*50*_ values were determined by analyzing the plot of the logarithm of the inhibitor concentration versus the percentage of activity remaining after inhibition, using the SPSS for Windows Version 11.5 (SPSS Inc., USA). The inhibition data were fitted to different models of enzyme inhibition (competitive, noncompetitive, and uncompetitive) by nonlinear least-squares regression analysis using GraphPad Prism 5 software (GraphPad Co. Ltd., USA) to calculate the *K*_*i*_ values.

## Results

### Kinetic analysis of STS

**Fig. 2:**
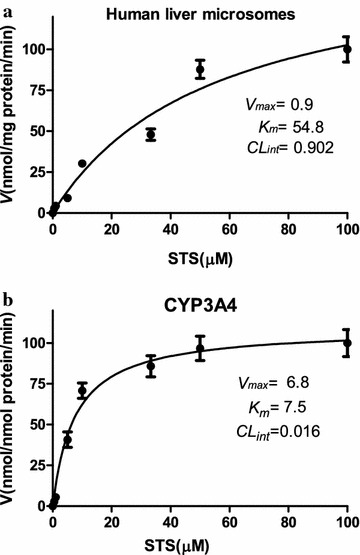
Concentration-velocity curves for STS metabolism after incubation with HLMs (**a**) and CYP3A4 (**b**). The *curves* were automatically fitted using nonlinear regression and the Michaelis–Menten equation, and the data were obtained in triple experiments

The metabolism of STS after incubation with HLMs and CYP3A4 was shown in Fig. 2a, b. The *K*_*m*_ and *V*_*max*_ values for HLMs and CYP3A4 were 54.8 ± 14.6 µM and 0.9 ± 0.1 nmol/mg protein/min, respectively, and 7.5 ± 1.4 µM and 6.8 ± 0.3 nmol/nmol P450/min, respectively. The in vitro *CL*_*int*_ values for STS with HLMs and CYP3A4 were 0.016 mL/mg protein/min and 0.902 mL/nmol P450/min, respectively.

### Specific CYP isoforms for the metabolism of STS

**Fig. 3:**
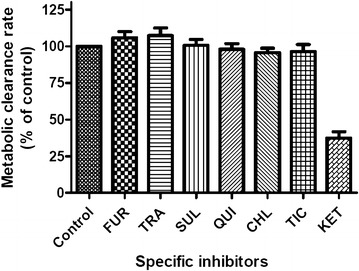
Effects of specific inhibitors on CYP-mediated STS (10 µM) metabolism in HLMs. The concentration of CHL was 5 µM, while that for the other specific inhibitors was 1 µM. Each *data point* represents the mean ± SD of triple determinations (n = 3). In the presence of KET (1 µM), the MCR of STS decreased to 27.28 % of the control value, while the other inhibitors had no significant inhibitory effects on the metabolism of STS. *FUR* furafylline, specific inhibitor of CYP1A2; *TRA* trans-2-phenylcyclopropylamine, specific inhibitor of CYP2A6; *SUL* sulfaphenazole, specific inhibitor of CYP2C9; *QUI* quinidine, specific inhibitor of CYP2D6; CHL: chlormethiazole, specific inhibitor of CYP2E1; *TIC*ticlopidine, specific inhibitor of CYP2C19; *KET* ketoconazole, specific inhibitor of CYP3A4

**Fig. 4:**
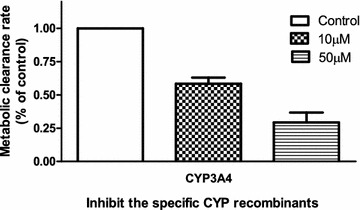
Effects on the MCR of STS under inhibition of CYP3A4 by KET (1 µM). STS (10 or 50 μM) was incubated with CYP3A4 and cofactors in the absence (control) or presence of KET. Each *point* represents the mean ± SD of triple incubations. The MCR of STS was significantly decreased compared with the control value for CYP3A4, for both concentrations of STS

The inhibitory effects of the CYP specific inhibitors on the MCR of STS in HLMs were shown in Fig. 3. The concentrations of FUR, TRA, SUL, QUI, TIC, and KET were 1 µM, while that of CHL was 5 µM. The concentrations were selected on the basis of previously reported *IC*_*50*_ or *K*_*i*_ values for the CYP isoforms to ensure adequate inhibitory selectivity, and maximal inhibitory potency [26–29]. In the presence of KET (1 µM), the MCR of STS decreased to 37.4 % of that of the control. However, the other inhibitors had no obvious inhibitory effects on the metabolism of STS. The effects of the screened enzymes were further confirmed with human recombinant CYPs using specific inhibitors, and the MCR of STS was decreased to 58.4 % (STS, 10 µM) and 29.4 % (STS, 50 µM) of the control value for CYP3A4 (Fig. 4), indicating that CYP3A4 was the major enzyme responsible for the metabolism of STS in vitro.

### Estimation of *IC*_*50*_ and *K*_*i*_ values

**Fig. 5:**
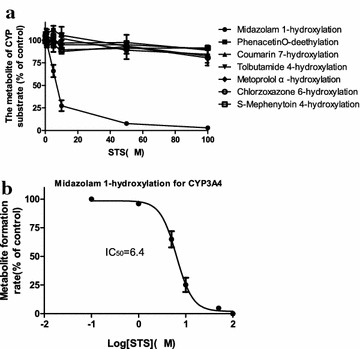
Effects of STS on the metabolic reactions of the seven CYP specific substrates in HLMs (**a**) and the representative *IC*_*50*_ *plots* of STS on midazolam 1-hydroxylation for CYP3A4 (**b**). Each *data point* represents the mean ± SD of triple determinations

The inhibitory effects of multiple concentrations of STS (1–100 µM) on the activity of each CYP isoform were determined by metabolism of a single concentration of isoform-specific probe with HLMs (or expressed CYPs, when needed). STS showed potent inhibition of CYP3A4 (midazolam 1-hydroxylation), with an *IC*_*50*_ of 6.4 µM. The inhibitory effects of STS on the activities of CYP1A2, CYP2A6, CYP2C9, CYP2D6, CYP2E1, and CYP2C19 were negligible (Fig. 5); therefore, we did not calculate the *IC*_*50*_ or *K*_*i*_ values for these enzymes related to STS.

## Discussion

According to Jeong et al. [30], *IC*_*50*_ values are qualitatively informative and helpful for addressing whether inhibition has occurred, but are of limited quantitative use because they can be influenced by the substrate concentration selected. Hence, it might not be accurate to use these parameters for quantitative prediction of drug interactions in vivo. Therefore, we performed additional experiments designed to estimate the *K*_*i*_ values. The preliminary inhibition data generated from a single probe substrate reaction were used to simulate the appropriate range of substrate and inhibitor concentrations for use in the construction of Dixon plots for inhibition of the CYP isoforms by STS in HLMs, from which precise *K*_*i*_ values were estimated.

**Fig. 6:**
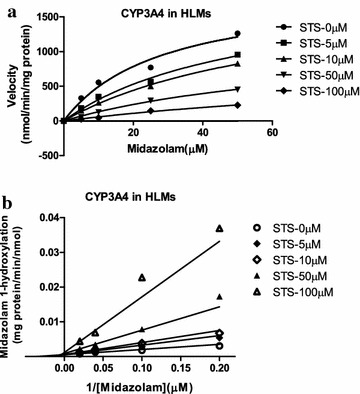
Non-linear regression and double reciprocal (Lineweaver–Burk) *plots* for direct inhibition of midazolam 1-hydroxylation by different concentrations of STS (0–100 μM) in HLM incubations (0.5 mg protein/mL). The inhibition of CYP3A4 activity by STS can be best-described as full competitive inhibition. Each *data point* is the mean of triple incubations

**Fig. 7:**
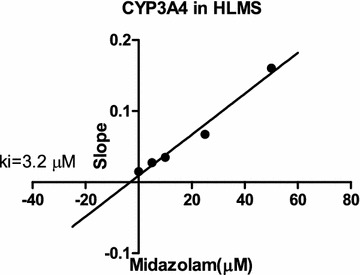
Secondary *plot* of CYP3A4 activity using the slopes of the primary Lineweaver–Burk *plots* versus concentrations of midazolam, illustrating the effects of STS on the metabolism of midazolam 1-hydroxylation in HLMs. Each *point* represents the mean of triple determinations

For CYP3A4, the *K*_*i*_ values were determined by midazolam as the probe substrate. Among all of the CYPs tested, CYP3A4 was the most sensitive to inhibition by STS (Table 2). Representative Dixon plots for the inhibition of CYP3A4 in HLMs were shown in Fig. 6. Visual inspection of the Dixon plots and further analysis of the parameters of the enzyme inhibition models suggested that the inhibition data fitted well to a competitive type of inhibition (Fig. 7). The *K*_*i*_ values estimated by a nonlinear regression model for competitive enzyme inhibition of CYP3A4-catalyzed midazolam 1-hydroxylation in HLMs were less than 5 µM (Table 2, Fig. 7).

According to Kong et al. [31], the potency of a test compound could be classified according to its *IC*_*50*_ values, as follows: potent, if *IC*_*50*_ is ≤20 μg/mL or ≤10 μM; moderate, if *IC*_*50*_ is 20–100 μg/mL or 10–50 μM; and weak, if *IC*_*50*_ is ≥100 μg/mL or ≥50 μM. Thus, STS was a potent inhibitor for CYP3A4 and a weak inhibitor for the other six CYPs tested in this study.

Although STS was a potent inhibitor of CYP3A4, the *IC*_*50*_ value of CYP3A4 for midazolam 1-hydroxylation was 26.6–79.7-fold higher and the *K*_*i*_ value was approximately 212-fold higher than that of KET in HLMs compared with a previous study [26] (Table 2), indicating that the inhibitory effect of STS on CYP3A4 was much less than that of KET.

Although the molecular structural difference between STS and tanshinone IIA is only the presence of a sulfonic acid group bond at the C-16 position [22], it resulted in a difference in inhibition of CYP activity. Wang et al. [32] reported that tanshinone IIA was a potent competitive CYP1A2 inhibitor with *K*_*i*_ values of 1.45 μM for pooled HLMs and 0.05 μM for a specific human CYP1A2 isoform, and a medium competitive inhibitor of CYP2C9. In this study, STS was a potent competitive CYP3A4 inhibitor with a *K*_*i*_ value of 3.2 μM for pooled HLMs, but was not an inhibitor of CYP1A2.

The CYP3A4 enzyme, one of the dominant CYP enzymes in both the liver and extrahepatic tissues such as the intestine, plays an important role in the oxidation of xenobiotics and contributes to the biotransformation of about 60 % of currently used therapeutic drugs [33]. Human CYP3A4 is one of the most abundant drug-metabolizing P450 isoforms in HLMs, and accounts for approximately 40 % of the total P450 activity [32]. Because STS was a potent inhibitor of CYP3A4 and given that CYP3A4 is responsible for the metabolism and disposition of a large number of currently used drugs, the potential herb–drug interactions of STS with drugs that were substrates of CYP3A4 or drugs with a narrow therapeutic index could not be negligible in the clinic. The present study supports the notion that STS is a substrate of CYP3A4 alone, while the issue of whether STS may be metabolized by other enzyme systems, such as more complex in vitro systems (hepatocytes for instance) that are not present (or functional in microsomes) remains unclear. Therefore, the present data do not support that STS would be a victim of drug–drug interactions (DDIs) by other CYP3A4 inhibitors, and further studies are required to elucidate this point.

Although STS in the form of injections is often used in clinical settings, pharmacokinetics studies in humans were lacking. Meanwhile, (Additional file 1) pharmacokinetics studies [15, 16] of STS in rats indicated that STS was widely distributed in most tissues after intravenous administration (2 mg/kg), and that it was mainly cleared via both the liver and kidney. The maximal STS concentration (>10 μg/mL) was found in the liver at 5 min after drug administration, subsequently declined progressively during 30 min, and then decreased quickly over time thereafter. STS could be determined at 12 and 4 h after drug administration in the liver and kidney, respectively. STS was hardly detected at 2 h after drug administration in other tissues [15, 16]. Therefore, co-administered medicines were suggested to be given at least 2 h after STS administration to avoid the risk of DDIs with STS.

Genetic variations in the *CYP3A4* gene may influence the level or function of the CYP3A4 protein, and more than 30 single nucleotide polymorphisms (SNPs) have been identified in the *CYP3A4* gene [34]. In previous studies, allelic variants in the gene encoding CYP3A4 were shown to affect enzyme activities, such as observations that variant CYP3A4 forms T363 M (<40 %) and T185S (<60 %) reduced testosterone 6β- and 2β-hydroxylase activities compared with the wild-type enzyme [35], while some SNPs in the *CYP3A4* gene such as CYP3A4*1B and CYP3A4*22 were reported to affect the pharmacokinetics of tacrolimus [36]. However, the issue of whether genetic polymorphisms of CYP3A4 can affect the DDIs of STS requires further study for clarification in the future.

## Conclusion

This in vitro study showed that STS mainly inhibited the activities of CYP3A4.

## Additional file

10.1186/s13020-016-0083-z The post-hoc test for the effects of the specific inhibitors on the MCR of STS.

## Abbreviations

STS: sodium tanshinone IIA sulfonate
CYPs: cytochrome P450 enzymes
HLMs: human liver microsomes
NADPH: β-nicotinamide adenine dinucleotide 2′-phosphate reduced tetrasodium salt hydrate
FUR: furafylline
TRA: trans-2-phenylcyclopropylamine hydrochloride
KET: ketoconazole
SUL: sulfaphenazole
QUI: quinidine
CHL: chlormethiazole hydrochloride
TIC: ticlopidine hydrochloride
MCR: metabolic clearance rate
