# Supplementary material for: Kinetics of cytochrome P450 enzymes for metabolism of sodium tanshinone IIA sulfonate in vitro
Source: Chin Med. 2016 Mar 22;11:11. doi: 10.1186/s13020-016-0083-z (PMC4802617; doi:10.1186/s13020-016-0083-z)
Supplement: Supplementary file 1 — 10.1186/s13020-016-0083-z The post-hoc test for the effects of the specific inhibitors on the MCR of STS. [file 13020_2016_83_MOESM1_ESM.docx]

One-way analysis of variance

|  | | | Sum of squares | df | mean square | F | significance |
| --- | --- | --- | --- | --- | --- | --- | --- |
| Between groups | （combination） | | 32544.023 | 7 | 4649.146 | 32.730 | .000 |
|  | Linear term | comparison | 14637.707 | 1 | 14637.707 | 103.048 | .000 |
|  |  | deviation | 17906.316 | 6 | 2984.386 | 21.010 | .000 |
| Within the group | | | 9091.014 | 64 | 142.047 |  |  |
| sum | | | 41635.038 | 71 |  |  |  |

Multiple comparison

|  | (I) VAR00001 | (J) VAR00001 | Mean Difference (I-J) | Standard error | significance | 95% confidence interval | |
| --- | --- | --- | --- | --- | --- | --- | --- |
|  |  |  |  |  |  | lower limit | upper limit |
| LSD | 1.00 | 2.00 | -5.80270 | 5.61836 | .306 | -17.0267 | 5.4213 |
|  |  | 3.00 | -7.28540 | 5.61836 | .199 | -18.5094 | 3.9386 |
|  |  | 4.00 | -.71994 | 5.61836 | .898 | -11.9439 | 10.5040 |
|  |  | 5.00 | 2.00724 | 5.61836 | .722 | -9.2167 | 13.2312 |
|  |  | 6.00 | 4.23427 | 5.61836 | .454 | -6.9897 | 15.4582 |
|  |  | 7.00 | 3.58499 | 5.61836 | .526 | -7.6390 | 14.8090 |
|  |  | 8.00 | 62.64233^*^ | 5.61836 | .000 | 51.4184 | 73.8663 |
|  | 2.00 | 1.00 | 5.80270 | 5.61836 | .306 | -5.4213 | 17.0267 |
|  |  | 3.00 | -1.48270 | 5.61836 | .793 | -12.7067 | 9.7413 |
|  |  | 4.00 | 5.08276 | 5.61836 | .369 | -6.1412 | 16.3067 |
|  |  | 5.00 | 7.80994 | 5.61836 | .169 | -3.4140 | 19.0339 |
|  |  | 6.00 | 10.03697 | 5.61836 | .079 | -1.1870 | 21.2609 |
|  |  | 7.00 | 9.38769 | 5.61836 | .100 | -1.8363 | 20.6117 |
|  |  | 8.00 | 68.44503^*^ | 5.61836 | .000 | 57.2211 | 79.6690 |
|  | 3.00 | 1.00 | 7.28540 | 5.61836 | .199 | -3.9386 | 18.5094 |
|  |  | 2.00 | 1.48270 | 5.61836 | .793 | -9.7413 | 12.7067 |
|  |  | 4.00 | 6.56546 | 5.61836 | .247 | -4.6585 | 17.7894 |
|  |  | 5.00 | 9.29264 | 5.61836 | .103 | -1.9313 | 20.5166 |
|  |  | 6.00 | 11.51967^*^ | 5.61836 | .044 | .2957 | 22.7436 |
|  |  | 7.00 | 10.87039 | 5.61836 | .057 | -.3536 | 22.0944 |
|  |  | 8.00 | 69.92773^*^ | 5.61836 | .000 | 58.7038 | 81.1517 |
|  | 4.00 | 1.00 | .71994 | 5.61836 | .898 | -10.5040 | 11.9439 |
|  |  | 2.00 | -5.08276 | 5.61836 | .369 | -16.3067 | 6.1412 |
|  |  | 3.00 | -6.56546 | 5.61836 | .247 | -17.7894 | 4.6585 |
|  |  | 5.00 | 2.72718 | 5.61836 | .629 | -8.4968 | 13.9511 |
|  |  | 6.00 | 4.95421 | 5.61836 | .381 | -6.2698 | 16.1782 |
|  |  | 7.00 | 4.30492 | 5.61836 | .446 | -6.9190 | 15.5289 |
|  |  | 8.00 | 63.36227^*^ | 5.61836 | .000 | 52.1383 | 74.5862 |
|  | 5.00 | 1.00 | -2.00724 | 5.61836 | .722 | -13.2312 | 9.2167 |
|  |  | 2.00 | -7.80994 | 5.61836 | .169 | -19.0339 | 3.4140 |
|  |  | 3.00 | -9.29264 | 5.61836 | .103 | -20.5166 | 1.9313 |
|  |  | 4.00 | -2.72718 | 5.61836 | .629 | -13.9511 | 8.4968 |
|  |  | 6.00 | 2.22703 | 5.61836 | .693 | -8.9969 | 13.4510 |
|  |  | 7.00 | 1.57775 | 5.61836 | .780 | -9.6462 | 12.8017 |
|  |  | 8.00 | 60.63509^*^ | 5.61836 | .000 | 49.4111 | 71.8591 |
|  | 6.00 | 1.00 | -4.23427 | 5.61836 | .454 | -15.4582 | 6.9897 |
|  |  | 2.00 | -10.03697 | 5.61836 | .079 | -21.2609 | 1.1870 |
|  |  | 3.00 | -11.51967^*^ | 5.61836 | .044 | -22.7436 | -.2957 |
|  |  | 4.00 | -4.95421 | 5.61836 | .381 | -16.1782 | 6.2698 |
|  |  | 5.00 | -2.22703 | 5.61836 | .693 | -13.4510 | 8.9969 |
|  |  | 7.00 | -.64928 | 5.61836 | .908 | -11.8733 | 10.5747 |
|  |  | 8.00 | 58.40806^*^ | 5.61836 | .000 | 47.1841 | 69.6320 |
|  | 7.00 | 1.00 | -3.58499 | 5.61836 | .526 | -14.8090 | 7.6390 |
|  |  | 2.00 | -9.38769 | 5.61836 | .100 | -20.6117 | 1.8363 |
|  |  | 3.00 | -10.87039 | 5.61836 | .057 | -22.0944 | .3536 |
|  |  | 4.00 | -4.30492 | 5.61836 | .446 | -15.5289 | 6.9190 |
|  |  | 5.00 | -1.57775 | 5.61836 | .780 | -12.8017 | 9.6462 |
|  |  | 6.00 | .64928 | 5.61836 | .908 | -10.5747 | 11.8733 |
|  |  | 8.00 | 59.05734^*^ | 5.61836 | .000 | 47.8334 | 70.2813 |
|  | 8.00 | 1.00 | -62.64233^*^ | 5.61836 | .000 | -73.8663 | -51.4184 |
|  |  | 2.00 | -68.44503^*^ | 5.61836 | .000 | -79.6690 | -57.2211 |
|  |  | 3.00 | -69.92773^*^ | 5.61836 | .000 | -81.1517 | -58.7038 |
|  |  | 4.00 | -63.36227^*^ | 5.61836 | .000 | -74.5862 | -52.1383 |
|  |  | 5.00 | -60.63509^*^ | 5.61836 | .000 | -71.8591 | -49.4111 |
|  |  | 6.00 | -58.40806^*^ | 5.61836 | .000 | -69.6320 | -47.1841 |
|  |  | 7.00 | -59.05734^*^ | 5.61836 | .000 | -70.2813 | -47.8334 |
| Tamhane | 1.00 | 2.00 | -5.80270 | 4.16942 | .998 | -24.8492 | 13.2438 |
|  |  | 3.00 | -7.28540 | 5.18761 | .998 | -30.9832 | 16.4124 |
|  |  | 4.00 | -.71994 | 3.96765 | 1.000 | -18.8447 | 17.4049 |
|  |  | 5.00 | 2.00724 | 3.94365 | 1.000 | -16.0079 | 20.0224 |
|  |  | 6.00 | 4.23427 | 2.95872 | .997 | -9.2816 | 17.7501 |
|  |  | 7.00 | 3.58499 | 4.79718 | 1.000 | -18.3292 | 25.4992 |
|  |  | 8.00 | 62.64233^*^ | 4.34824 | .000 | 42.7789 | 82.5057 |
|  | 2.00 | 1.00 | 5.80270 | 4.16942 | .998 | -13.2438 | 24.8492 |
|  |  | 3.00 | -1.48270 | 6.65548 | 1.000 | -26.5165 | 23.5511 |
|  |  | 4.00 | 5.08276 | 5.75555 | 1.000 | -16.3849 | 26.5505 |
|  |  | 5.00 | 7.80994 | 5.73903 | .997 | -13.5987 | 29.2186 |
|  |  | 6.00 | 10.03697 | 5.11254 | .866 | -9.4276 | 29.5016 |
|  |  | 7.00 | 9.38769 | 6.35586 | .992 | -14.3962 | 33.1716 |
|  |  | 8.00 | 68.44503^*^ | 6.02422 | .000 | 45.9782 | 90.9119 |
|  | 3.00 | 1.00 | 7.28540 | 5.18761 | .998 | -16.4124 | 30.9832 |
|  |  | 2.00 | 1.48270 | 6.65548 | 1.000 | -23.5511 | 26.5165 |
|  |  | 4.00 | 6.56546 | 6.53097 | 1.000 | -18.1057 | 31.2367 |
|  |  | 5.00 | 9.29264 | 6.51642 | .995 | -15.3378 | 33.9230 |
|  |  | 6.00 | 11.51967 | 5.97205 | .892 | -11.8951 | 34.9344 |
|  |  | 7.00 | 10.87039 | 7.06571 | .987 | -15.5025 | 37.2432 |
|  |  | 8.00 | 69.92773^*^ | 6.76894 | .000 | 44.5441 | 95.3114 |
|  | 4.00 | 1.00 | .71994 | 3.96765 | 1.000 | -17.4049 | 18.8447 |
|  |  | 2.00 | -5.08276 | 5.75555 | 1.000 | -26.5505 | 16.3849 |
|  |  | 3.00 | -6.56546 | 6.53097 | 1.000 | -31.2367 | 18.1057 |
|  |  | 5.00 | 2.72718 | 5.59416 | 1.000 | -18.1289 | 23.5832 |
|  |  | 6.00 | 4.95421 | 4.94937 | 1.000 | -13.7887 | 23.6971 |
|  |  | 7.00 | 4.30492 | 6.22536 | 1.000 | -19.0613 | 27.6711 |
|  |  | 8.00 | 63.36227^*^ | 5.88638 | .000 | 41.3819 | 85.3427 |
|  | 5.00 | 1.00 | -2.00724 | 3.94365 | 1.000 | -20.0224 | 16.0079 |
|  |  | 2.00 | -7.80994 | 5.73903 | .997 | -29.2186 | 13.5987 |
|  |  | 3.00 | -9.29264 | 6.51642 | .995 | -33.9230 | 15.3378 |
|  |  | 4.00 | -2.72718 | 5.59416 | 1.000 | -23.5832 | 18.1289 |
|  |  | 6.00 | 2.22703 | 4.93015 | 1.000 | -16.4317 | 20.8858 |
|  |  | 7.00 | 1.57775 | 6.21009 | 1.000 | -21.7411 | 24.8966 |
|  |  | 8.00 | 60.63509^*^ | 5.87022 | .000 | 38.7102 | 82.5600 |
|  | 6.00 | 1.00 | -4.23427 | 2.95872 | .997 | -17.7501 | 9.2816 |
|  |  | 2.00 | -10.03697 | 5.11254 | .866 | -29.5016 | 9.4276 |
|  |  | 3.00 | -11.51967 | 5.97205 | .892 | -34.9344 | 11.8951 |
|  |  | 4.00 | -4.95421 | 4.94937 | 1.000 | -23.6971 | 13.7887 |
|  |  | 5.00 | -2.22703 | 4.93015 | 1.000 | -20.8858 | 16.4317 |
|  |  | 7.00 | -.64928 | 5.63621 | 1.000 | -22.4978 | 21.1993 |
|  |  | 8.00 | 58.40806^*^ | 5.25939 | .000 | 38.2842 | 78.5319 |
|  | 7.00 | 1.00 | -3.58499 | 4.79718 | 1.000 | -25.4992 | 18.3292 |
|  |  | 2.00 | -9.38769 | 6.35586 | .992 | -33.1716 | 14.3962 |
|  |  | 3.00 | -10.87039 | 7.06571 | .987 | -37.2432 | 15.5025 |
|  |  | 4.00 | -4.30492 | 6.22536 | 1.000 | -27.6711 | 19.0613 |
|  |  | 5.00 | -1.57775 | 6.21009 | 1.000 | -24.8966 | 21.7411 |
|  |  | 6.00 | .64928 | 5.63621 | 1.000 | -21.1993 | 22.4978 |
|  |  | 8.00 | 59.05734^*^ | 6.47457 | .000 | 34.8748 | 83.2399 |
|  | 8.00 | 1.00 | -62.64233^*^ | 4.34824 | .000 | -82.5057 | -42.7789 |
|  |  | 2.00 | -68.44503^*^ | 6.02422 | .000 | -90.9119 | -45.9782 |
|  |  | 3.00 | -69.92773^*^ | 6.76894 | .000 | -95.3114 | -44.5441 |
|  |  | 4.00 | -63.36227^*^ | 5.88638 | .000 | -85.3427 | -41.3819 |
|  |  | 5.00 | -60.63509^*^ | 5.87022 | .000 | -82.5600 | -38.7102 |
|  |  | 6.00 | -58.40806^*^ | 5.25939 | .000 | -78.5319 | -38.2842 |
|  |  | 7.00 | -59.05734^*^ | 6.47457 | .000 | -83.2399 | -34.8748 |
| *. The significance level difference for the average is 0.05。 | | | | | | | |
